# Artificial Potential Field Based Trajectory Tracking for Quadcopter UAV Moving Targets

**DOI:** 10.3390/s24041343

**Published:** 2024-02-19

**Authors:** Cezary Kownacki

**Affiliations:** Department of Industrial Processes Automation, Faculty of Mechanical Engineering, Bialystok University of Technology, Wiejska St. 45C, 15-351 Bialystok, Poland; c.kownacki@pb.edu.pl; Tel.: +48-571-443-054

**Keywords:** quadcopter UAV, artificial potential field, trajectory tracking, holonomic UAV

## Abstract

The trajectory or moving-target tracking feature is desirable, because it can be used in various applications where the usefulness of UAVs is already proven. Tracking moving targets can also be applied in scenarios of cooperation between mobile ground-based and flying robots, where mobile ground-based robots could play the role of mobile landing pads. This article presents a novel proposition of an approach to position-tracking problems utilizing artificial potential fields (APF) for quadcopter UAVs, which, in contrast to well-known APF-based path planning methods, is a dynamic problem and must be carried out online while keeping the tracking error as low as possible. Also, a new flight control is proposed, which uses roll, pitch, and yaw angle control based on the velocity vector. This method not only allows the UAV to track a point where the potential function reaches its minimum but also enables the alignment of the course and velocity to the direction and speed given by the velocity vector from the APF. Simulation results present the possibilities of applying the APF method to holonomic UAVs such as quadcopters and show that such UAVs controlled on the basis of an APF behave as non-holonomic UAVs during 90° turns. This allows them and the onboard camera to be oriented toward the tracked target. In simulations, the AR Drone 2.0 model of the Parrot quadcopter is used, which will make it possible to easily verify the method in real flights in future research.

## 1. Introduction

Flight through a planned path and trajectory tracing is the most ordinary capability of modern unmanned aerial vehicles. Plenty of research focuses on different approaches to the problem of finding the optimal path planning and tracking, considering movement among obstacles in an uncertain environment, and exploring the possibilities of swarm flights in complex scenarios. One useful technique to achieve smooth and optimal path routing is a method based on the idea of artificial potential fields and their variations. Their popularity is due to their fundamental rules, which define a velocity vector field over the considered area, derived from gradients of the potential function, which reaches minimums at targets and maximums at obstacles. A combination of repulsive and attractive forces and related velocity vectors push robots away from obstacles and toward targets.

There are plenty of articles dedicated to the problem of planning UAV trajectories. The main issue to be solved during the process of path planning is the local minimum, where a robot could be trapped while it moves towards the target. A local minimum is usually the effect of a sum of several potential fields located around obstacles, waypoints, and final targets. In [1], a new planning algorithm is split into local and global horizons by applying deterministic annealing. Introducing a temperature parameter improves the efficiency of obstacle avoidance. The annealing and tempering strategies make a robot able to avoid local minimums. Another solution for discarding the weakness of a traditional potential field, which involves falling into local errors, is to combine an artificial potential field with a rapidly exploring random tree method. This kind of approach is described in [2], where the called PF-RRT method accelerates the process of searching through the tree and takes full advantage of the potential field. The quality of path generation is also improved by using the principle of triangle inequality. The drawbacks in global optimization capacity and speed are also a barrier to implementing artificial potential fields in practical applications. To address this, a new stimulating rotating repulsive force, defined in a new form of potential function, with higher effectiveness and practicability, was presented in [3]. The rotating artificial potential field (R-APF) also prevents a UAV from becoming trapped in a local minimum. In [4], an improved artificial-potential-field method is demonstrated. The authors introduce a collision-risk assessment mechanism and virtual subtargets, respectively, to avoid unreasonable obstacle avoidance and solve the problems of local minimums and unreachable targets. Artificial-potential-field methods are also applicable in path planning of multi-UAV formations and are challenging for non-holonomic UAVs. The main disadvantage of APFs is the lack of initial constraints on the UAV’s heading, which can cause a fall into a local minimum or target unreachability. Therefore, in [5], a piecewise-potential-field-based method of path planning is formulated. A suitable design of a piecewise potential function defining a potential field vector that meets the kinematic constraints of the UAV solves the problem of path planning in different flight states, and an additional potential field vector protects each formation member from getting stuck in the local minimum. A combination of attractive and repulsive forces is given in [6] to achieve formation obstacle avoidance. Traditional artificial potential fields may also fail in the case of fixed-wing UAVs due to a limited turn radius. Even the global minimum can become unreachable, and applying such APF methods results in the instability of formation flights, as proven in [7]. The presented local and asymmetrical potential field (LA-PF) allows for its application to formation flights, since it can be considered as a candidate for a Lyapunov function, which has a global minimum and defines the velocity vector field in such a way that position control is achieved by speed control along the direction of the tracked position movement and by heading control in the longitudinal direction. Violent heading changes near the tracked position do not cause a rapid turn with a minimal radius. The local and asymmetrical potential function is also a framework for future research on nonlinear PID control loops [8], where the definition of LA-PF is extended with the integral and derivative terms of PID control. Therefore, position-tracking control becomes more resistant to disturbances like wind gusts, and steady-state tracking errors can be minimized. Since artificial potential fields allow the creation of a spatial distribution of repulsive vectors, they are commonly used in obstacle avoidance by UAVs, including dynamic obstacles. Optimal path planning among static and dynamic obstacles is presented in [9]. The method is implemented into the Parrot AR Drone 2.0 Quadcopter UAV model. Results are obtained from a simulation in Gazebo Simulator by Robot Operating System (ROS). Path planning in unknown environments with an aerial robot is presented in [10]. The conventional artificial potential field is used, but flight paths are modified in real time depending on a map of the surroundings gathered by a 3D LiDAR sensor. The rotation-based component of the APF helps to avoid local minimums. In [11], the presented method of path planning transforms the discrete path model into a continuous one in order to realize path tracking control based on sliding mode control (SMC). An obstacle avoidance strategy based on artificial potential field and considering a possible sudden motor loss-of-effectiveness fault of the UAV can be found in [12].

Among all the research, it is difficult to find a work dedicated to the problem of tracking a moving point, which would be solved by an artificial potential field for a quadcopter. The problem is similar to that of formation flight but with no repulsive relations between UAVs inside the formation. In the case of a quadcopter, the main issue to be solved is also to design control that will guide a holonomic vehicle according to the velocity vector from the potential field, keeping its speed, position, and course as close to those resulting from the free movement of the reference position. Only then is it possible to track a random unknown trajectory in real time, not only along a predefined planned path, and this is the main significance of the work cited above in contrast to those based on path planning [13] (even if a short time horizon of the ground target movement prediction is available [14]) and on path optimization: particle swarm optimization and pigeon-inspired optimization. The present work presents a method that applies an artificial-potential-field approach and modified flight control for quadcopters to achieve the tracking of a freely moving point with unknown dynamics. Thus, in such conditions, MPC (model predictive control) [15] or Kalman filter [16] based methods cannot be applied, because the estimation or prediction of the target’s movement within the time horizon is not possible, or the model of the target is not similar to the UAV in contrast to [17], where sliding mode control is applied. The proposed method can also be used in applications where a virtual moving point is controlled by a ground station to achieve complex shapes of trajectories with the use of any local positioning system. The research is performed in MATLAB by using the AR Drone 2.0 model. To present the effectiveness of tracking the control, three different reference trajectory shapes are used in simulations, which include sharp turns as the most critical section to be tracked. In contrast to the presented simulation results, in [18], the AR Drone 2.0 model with Gazebo software was used to plan flight paths with cubic polynomials and Bezier curves. The presented method can also be a source of training data for neural networks, and this will be an aim of future work, so this is another advantage. There are four sections following the introduction in this work. The next section is about quadcopter dynamics.

## 2. Quadcopter Dynamics (AR Drone)

**Figure 1:**
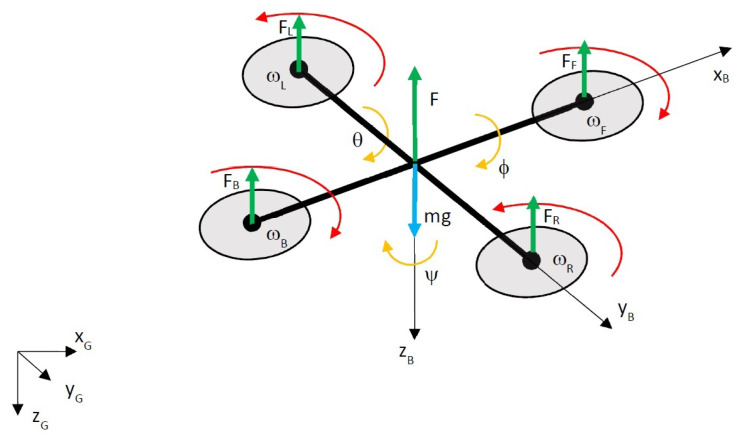
Dynamic model of quadcopter, $F_{F}$, $F_{B}$, $F_{L}$, $F_{R}$—thrusts of motors (subscripts: F—forward, B—back, R—right, L—left), F—total thrust, mg—force of gravity, $\omega_{F}$, $\omega_{B}$, $\omega_{R}$, $\omega_{L}$—angular rates of each motor, ϕ—roll angle, θ—pitch angle, Ψ—yaw angle, $x_{B}$, $y_{B}$, $z_{B}$—axes of body frame, $x_{G}$, $y_{G}$, $z_{G}$—axes of global reference frame.

Mathematical modeling of a quadcopter assumes that its body and propellers are rigid and symmetric and that it has 6 degrees of freedom (6 DoF) ([19]). Equations of motion use two reference frames, i.e., B—body frame and G—global frame, which can be considered as inertial. Figure 1 presents a dynamic model of a quadcopter.

The relationship between the frames of reference is given by rotation matrix *R* from Equation (1).

(1) $$R=\left[\begin{array}{ccc} \cos(\phi )*\cos(\Psi )-\cos(\theta )*\sin(\phi )*\sin(\Psi ) & -\cos(\Psi )*\sin(\phi )-\cos(\phi )*\cos(\theta )*\sin(\Psi ) & \sin(\theta )*\sin(\Psi )\\ \cos(\theta )*\cos(\Psi )*\sin(\phi )+\cos(\phi )*\sin(\Psi ) & \cos(\phi )*\cos(\theta )*\cos(\Psi )-\sin(\phi )*\sin(\Psi ) & -\cos(\Psi )*\sin(\theta )\\ \sin(\phi )*\sin(\theta ) & \cos(\phi )*\sin(\theta ) & \cos(\theta ) \end{array}\right]$$

where ϕ, θ, and Ψ are the roll, pitch, and yaw angles, respectively, i.e., the angles between the axes of the body frame and the axes of the inertial frame.

The equations of motion are defined in the inertial frame. The quadcopter’s acceleration is the result of thrust, gravity, and linear friction. Thus, the quadcopter’s linear motion is defined as follows:

(2) $$m\ddot{x}=\left[\begin{array}{c} 0\\ 0\\ -mg \end{array}\right]-R\cdot T_{B}-F_{D}$$

with *m*—mass of the quadcopter, *g*—acceleration of gravity, $\ddot{x}$—linear acceleration of the quadcopter, *R*—rotation matrix (Equation (1), $T_{B}$—thrust in the body frame (Equation (3)), and $F_{D}$—drag force (Equation (5)).

Thrust in the body frame can be obtained from:

(3) $$T_{B}=\left[\begin{array}{c} 0\\ 0\\ F_{R}+F_{L}+F_{F}+F_{B} \end{array}\right]=k\cdot \left[\begin{array}{c} 0\\ 0\\ \omega_{R}^{2}+\omega_{L}^{2}+\omega_{F}^{2}+\omega_{B}^{2} \end{array}\right]$$

with $\omega_{i}$—angular speed of the i-th motor and *k*—constant dependent on the use of a specific motor and propeller.

The drag force on each axis of the inertial frame can be defined by the equation for the frictional force taken from fluid dynamics:

(4) $$F_{d}=\frac{1}{2}\cdot \rho \cdot C_{D}\cdot A\cdot V^{2}$$

with ρ—the fluid’s (air’s) density, *A*—reference area (propeller cross section), $C_{D}$—dimensionless constant, and *V*—linear speed.

Equation (4) can be simplified for the purpose of modeling the quadcopter. The drag forces in the inertial frame are as follows:

(5) $$F_{D}=\left[\begin{array}{c} -k_{d}\cdot \dot{x}\\ -k_{d}\cdot \dot{y}\\ -k_{d}\cdot \dot{z} \end{array}\right]$$

with $k_{d}$—drag coefficient, $\dot{x}$, $\dot{y}$, and $\dot{z}$—linear speeds on each axis of the inertial frame.

Euler’s equations for rigid body dynamics are used to derive the rotational equations of motion.

(6) $$I\cdot \dot{\omega }+\omega \times \left(I\cdot \omega \right)=\tau_{B}$$

with *I*—inertial matrix, ω—angular velocity vector, and τ—vector of external torques in the body frame.

Because the symmetrical rigid body of the quadcopter can be modeled as two uniform rods crossed at the origin of the body frame, having points of mass at each end (motors), the inertial matrix is given by:

(7) $$I=\left[\begin{array}{ccc} I_{xx} & 0 & 0\\ 0 & I_{yy} & 0\\ 0 & 0 & I_{zz} \end{array}\right]$$

where $I_{xx}$, $I_{yy}$, $I_{yy}$ are the inertia on each axis.

The vector of external torques is as follows:

(8) $$\tau =\left[\begin{array}{c} \tau_{\phi }\\ \tau_{\theta }\\ \tau_{\Psi } \end{array}\right]=\left[\begin{array}{c} k\cdot (\omega_{R}^{2}\cdot L-\omega_{L}^{2}\cdot L)\\ k\cdot (\omega_{F}^{2}\cdot L-\omega_{B}^{2}\cdot L)\\ b\cdot (\omega_{R}^{2}+\omega_{L}^{2}-\omega_{F}^{2}-\omega_{B}^{2}) \end{array}\right]$$

where *b* is an appropriately dimensioned constant (from the definition of drag-induced torque), and *k* is a constant dependent on the use of a specific motor and propeller.

Equation (6) can be rewritten as the following form:

(9) $$\dot{\omega }=I^{-1}\cdot \left(\tau_{B}-\omega \times \left(\omega \cdot I\right)\right)$$

Substituting the inertial matrix (Equation (7)) and the vector of external torques (Equation (8)) into Equation (9), we finally obtain a rotational equation of motion:

(10) $$\dot{\omega }=\left[\begin{array}{c} \tau_{\phi }\cdot I_{xx}^{-1}\\ \tau_{\theta }\cdot I_{yy}^{-1}\\ \tau_{\Psi }\cdot I_{zz}^{-1} \end{array}\right]-\left[\begin{array}{c} \frac{I_{yy}-I_{zz}}{I_{xx}}\cdot \omega_{y}\cdot \omega_{z}\\ \frac{I_{zz}-I_{xx}}{I_{yy}}\cdot \omega_{x}\cdot \omega_{z}\\ \frac{I_{xx}-I_{yy}}{I_{zz}}\cdot \omega_{x}\cdot \omega_{y} \end{array}\right]$$

Equations (2) and (10) create a dynamic model of the quadcopter, which can be used in numerical simulations. In our work, we applied a ready model from the AR Drone 2.0 library for MATLAB/SIMULINK R2022a.

## 3. Artificial Potential Field and Quadcopter Control

In trajectory-tracking flight scenarios, only an attraction artificial potential field is required. In such cases, the minimum of the potential function is located at a target point to be tracked by the quadcopter. The attraction potential field must be symmetrical with respect to the minimum, and the most popular form is given by the equation [7]:

(11) $$U\left(x_{R},y_{R},z_{R}\right)=\left({\left(x_{R}-x_{T}\right)}^{2}+{\left(y_{R}-y_{T}\right)}^{2}+{\left(z_{R}-z_{T}\right)}^{2}\right)$$

where $x_{R}$, $y_{R}$, and $z_{R}$ are the coordinates of the robot’s (quadcopter’s) position, and $x_{T}$, $y_{T}$, and $z_{T}$ are the coordinates of the point to be tracked.

The potential function’s gradient (11) is as follows:

(12) $$\nabla U\left(x_{R},y_{R},z_{R}\right)=\left[\begin{array}{c} 2\cdot \left(x_{R}-x_{T}\right)\\ 2\cdot \left(y_{R}-y_{T}\right)\\ 2\cdot \left(z_{R}-z_{T}\right) \end{array}\right]$$

The definition of a velocity vector field that includes a saturation of the relative speed, i.e., the maximum length of the gradient (12) cannot be greater than the saturation value of $V_{max}$, is given by:

(13) $$V\left(x_{R},y_{R},z_{R}\right)=\{\begin{array}{c} -\nabla U\left(x_{R}.y_{R},z_{R}\right)\;\mathrm{for}\left|\nabla U(x_{R}.y_{R},z_{R})\right|\leq V_{max}\\ -\frac{V_{max}}{\left|\nabla U(x_{R}.y_{R},z_{R})\right|}\cdot \nabla U\left(x_{R}.y_{R},z_{R}\right)\;\mathrm{for}\left|\nabla U(x_{R}.y_{R},z_{R})\right|>V_{max} \end{array}$$

with $V_{max}$—the saturation value of the $\nabla U(x_{R}.y_{R},z_{R})$ gradient’s length and $\left|\nabla U(x_{R}.y_{R},z_{R})\right|$—the length of the gradient from (12).

**Figure 2:**
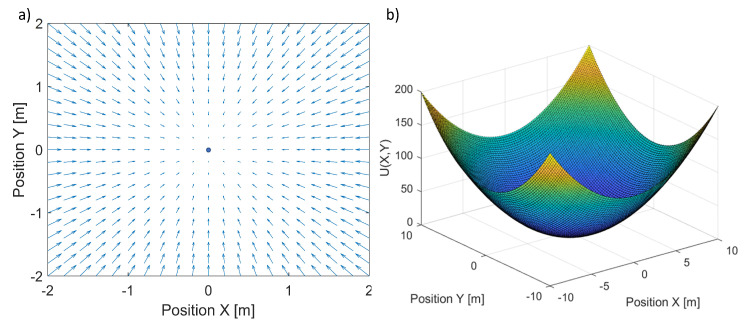
Plot of an artificial potential field for the 2D case (**a**) and the related field of velocity vectors (**b**).

Figure 2 presents a plot of the artificial potential function in (11) for the 2D case and a corresponding field of velocity vectors (13). The point at the center of Figure 2a is the minimum of the potential function. In this case, the field of velocity vectors is defined in a local coordinate frame fixed to the minimum point. Thus, the velocity vectors’ orientation and length come from the geometrical relationship between the minimum point and points in its surroundings. These velocity vectors guide the quadcopter toward the potential function’s minimum point, which can be considered stationary.

**Figure 3:**
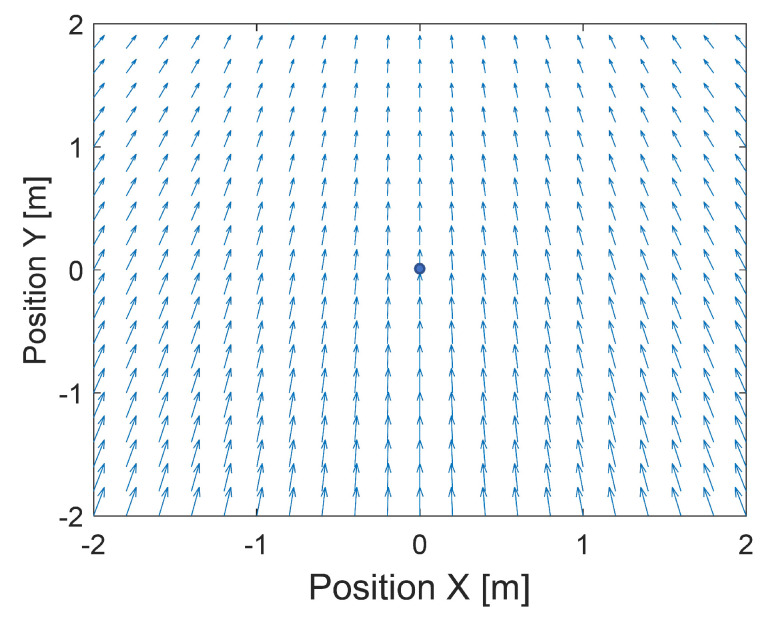
Plot of the superposition of the field of velocity vectors 13 and vector $V_{TR}$.

But suppose the minimum is moving with a velocity vector $V_{TR}$. In such case, the field of velocity vectors that guide the quadcopter should be considered as a superposition of the field of velocity vectors (13) and the vector $V_{TR}$. This superposition can be considered as the field of velocity vectors in the global frame, and it looks like in Figure 3.

A control velocity vector that can be applied to the controlled quadcopter’s trajectory is as follows:

(14) $$V_{C}\left(x_{R},y_{R},z_{R}\right)=V_{T}+V\left(x_{R},y_{R},z_{R}\right)$$

According to Equation (14), the control velocity vector $V_{C}$ is a combination of two independent control rules. In particular, the vector *V* based on the gradient of the potential function is responsible for minimizing the tracking error, and vector $V_{T}$ is responsible for the synchronized movement of the tracked point and the quadcopter. Vector $V_{T}$ can be determined simply as a derivative of the tracked point position.

**Figure 4:**
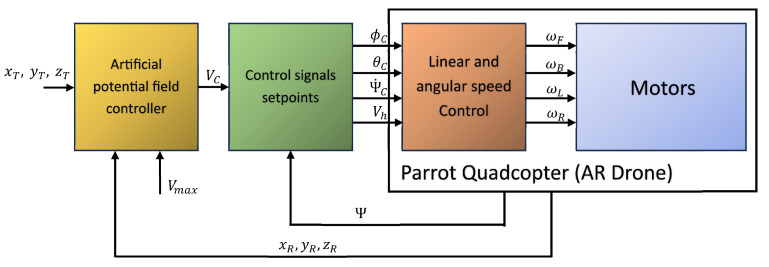
A diagram of the structure of tracking control with the artificial potential field method applied and used signals flow.

To use vector $V_{C}$ as a control input, it is necessary to design control laws that transform it into typical quadcopter control inputs, i.e., roll and pitch angles, yaw rate, and vertical velocity. A general diagram of the control system and signal flow used for quadcopter control is shown in Figure 4.

The inputs for the artificial-potential-field block are coordinates $x_{T}$, $y_{T}$, and $z_{T}$ of the point to be tracked, the coordinates of the actual quadcopter position, i.e., $x_{R}$, $y_{R}$, and $z_{R}$, and $V_{max}$, the saturation value of the $\nabla U(x_{R}.y_{R},z_{R})$ gradient’s length as a constant coefficient. The subsequent block in the control diagram calculates control signals of roll $\Phi_{C}$, pitch $\theta_{C}$, rate of yaw $\Psi_{C}$, and vertical speed $V_{h}$ based on velocity vector $V_{C}$ and the quadcopter’s actual yaw (heading) Ψ. The equations implemented in this block are given below. The block of a Parrot quadcopter’s model was taken from the AR Drone 2.0 MATLAB toolbox.

The quadcopter’s linear speeds in the forward and longitudinal directions are achieved, respectively, by the control of pitch and roll angles. Thus, desired roll and pitch angle values should be derived from vector $V_{C}$. To do this, it is necessary to determine the bearing to the tracked point given in the quadcopter’s body frame. Thus, vector $V_{C}$ must be rotated in the *z*-axis of the global coordinate frame by the quadcopter’s current heading angle Ψ. Next, the *x*-axis and *y*-axis components of rotated vector $V_{Cr}$ can be used to determine the required bearing. The rotation is defined by Equation (15) and the bearing by Equation (16).

(15) $$V_{Cr}=\left[\begin{array}{ccc} cos(\Psi ) & sin(\Psi ) & 0\\ -sin(\Psi ) & cos(\Psi ) & 0\\ 0 & 0 & 1 \end{array}\right]\cdot V_{C}$$

where Ψ is the quadcopter’s current heading angle, and $V_{C}$ is the velocity vector from Equation (14).

(16) $$\Psi_{B}=atan2\left(V_{Cr}\left(y\right),V_{Cr}\left(x\right)\right)$$

where $\Psi_{B}$ is the bearing to the tracked point, and $V_{Cr}\left(x\right)$ and $V_{Cr}\left(y\right)$ are the components of vector $V_{Cr}$ (Equation (14)).

Having vector $V_{Cr}$ and bearing $\Psi_{B}$, set points for the roll and pitch control loop can be obtained. The AR Drone 2.0 model of the Parrot quadcopter defines a range of desired roll and pitch angles from −1 to 1, which corresponds, respectively, to a range of longitudinal linear speed from −3.78 to 3.78 m/s and of transverse linear speed from −2.88 to 2.88 m/s. The resultant maximum horizontal speed of the Parrot quadcopter is about 4.75 m/s. The maximum vertical speed is about 0.88 m/s, which corresponds to control signal $V_{h}=1$. Calculations of desired roll and pitch angles should consider this range. The equations defining desired roll and pitch angles are as follows:

(17) $$\phi_{C}=\frac{\left|V_{Cr}\left(x,y\right)\right|}{2.88}\cdot sin\left(\Psi_{B}\right)$$

(18) $$\theta_{C}=\frac{\left|V_{Cr}\left(x,y\right)\right|}{3.78}\cdot cos\left(\Psi_{B}\right)$$

where $\Psi_{B}$ is the bearing to the tracked point, and $\left|V_{Cr}\left(x,y\right)\right|$ is the length of the x-y plane projection of vector $V_{Cr}$, limited to a range of <0,1.0>.

The ratios of $\left|V_{Cr}\left(x,y\right)\right|$ and 2.88 for $\phi_{C}$ and of $\left|V_{Cr}\left(x,y\right)\right|$ and 3.78 for $\theta_{C}$ determine the gain magnitudes of longitudinal and traverse speed control. In the case of our research, the range of $\left|V_{Cr}\left(x,y\right)\right|$ was <0,1.0>, thus these speed values were limited to 1 m/s. These gains can be changed relative to the maximum horizontal speed of the tracked point.

The vertical speed of the quadcopter $V_{h}$ is just the *z*-axis component of vector $V_{C}$, limited to the range <−1.0,1.0>.

(19) $$V_{h}=\{\begin{array}{c} k_{V_{h}}\cdot V_{C}\left(z\right)\;\mathrm{for}\;k_{V_{h}}\cdot V_{C}\left(z\right)\leq 1\cap k_{V_{h}}\cdot V_{C}\left(z\right)\geq -1)\\ 1\;\mathrm{for}\;k_{V_{h}}\cdot V_{C}\left(z\right)>1)\\ -1\;\mathrm{for}\;k_{V_{h}}\cdot V_{C}\left(z\right)<-1) \end{array}$$

where $k_{V_{h}}$ is the gain coefficient which regulates the climb rate.

The rate of yaw, which makes the quadcopter rotate around the *z*-axis of the global frame, depends on the heading error, i.e., the difference between the quadcopter’s actual heading Ψ and the heading obtained from vector $V_{C}$. Therefore, the equation for the yaw rate signal is as follows:

(20) $${\dot{\Psi }}_{C}=k_{\dot{\Psi }}\cdot \frac{\left(atan2(V_{C}\left(y\right),V_{C}\left(x\right))-\Psi \right)}{\pi }$$

with $k_{\dot{\Psi }}$-gain coefficient, Ψ-quadcopter’s actual heading, $V_{C}\left(y\right)$, $V_{C}\left(x\right)$-components of vector $V_{C}$, and $\left(atan2\left(V_{C}\left(y\right),V_{C}\left(x\right)\right)-\Psi \right)$-heading error in range <−π,π>.

In the AR Drone 2.0 model, all control signals’ ranges (i.e., $V_{h}$, $\phi_{C}$, $\theta_{C}$, and ${\dot{\Psi }}_{C}$) are from −1 to 1, thus the heading error in Equation (20) must be divided by π.

Finally, all of the quadcopter’s required control signals, which are obtained from velocity vector $V_{C}$, are given by Equations (15)–(20). The next step of research was to design trajectories to be tracked by the quadcopter (Parrot) and to carry out simulations visualizing the possibilities of the proposed method, which used the designed control based on the artificial potential field.

## 4. Simulation Scenarios and Results

**Figure 5:**
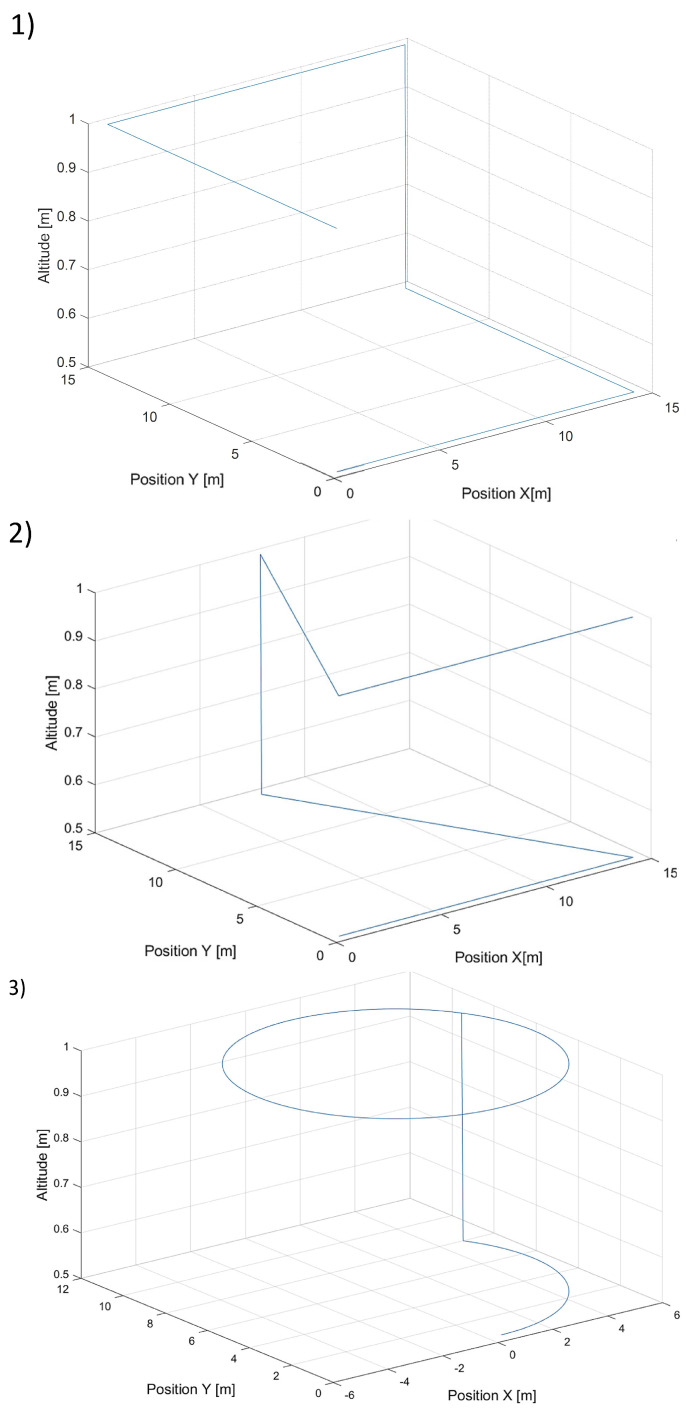
Plot of three trajectories of the point to be tracked by the quadcopter with (**1**)—a rectangular horizontal projection, (**2**)—a triangular horizontal projection, and (**3**)—a circular horizontal projection.

To verify the effectiveness and control quality of the designed tracking control, three different shapes of trajectories were designed. Each of them included altitude changes and different turn angles. The point to be tracked was moving along those trajectories with a constant speed, which was 0.7 m/s. In each simulated flight scenario, there was a step change in altitude of about 0.5 m to observe vertical control possibilities (at the 60th s of simulation). The movement of the tracked point started after a take-off command with a delay of 20 s. The delay allowed the UAV to perform the take-off phase, during which tracking control was not active. All reference trajectories used in the simulations are presented in Figure 5.

In all flight scenarios, the tracked point’s initial position was located in the global frame at coordinates x = 0.5, y = 0.5, and z = 0.5, and the quadcopter’s initial position was at x = y = x = 0.0. Therefore, the initial tracking error was the same each time and was about 0.86 m. The parameters impacting the flight trajectories we wanted to assess were the saturation value of the gradient’s length $V_{max}$ and gain coefficients $k_{\dot{\Psi }}$ and $k_{V_{h}}$. In simulations, we used the following values of the gradient length’s saturation $V_{max}$ = {0.7 m/s, 1.4 m/s, 2.1 m/s, 3.78 m/s}. Because $V_{max}$ can be treated as the saturation of the relative speed, the value of 3.78 m/s was the maximum relative speed (horizontal and vertical). The value of 4.75 was the maximum horizontal speed for the Parrot quadcopter corresponding to maximum roll and pitch angles. Still, the horizontal speed was limited to 1 m/s in the longitudinal and transverse directions, respectively, in Equations (17) and (18) by setting the limit of $\left|V_{Cr}\left(x,y\right)\right|$ to <0,1.0>. Thus, $V_{max}$ impacted vertical speed most of all, and its sum with the speed of the tracked point $V_{T}$ was lower than the maximum speed of the quadcopter. The values of $k_{\dot{\Psi }}$ and $k_{V_{h}}$ used in the investigation were 0.5, 1.0, 2.0, and 2.5 for $k_{\dot{\Psi }}$ and 0.1, 0.5, 1.0, and 1.5 for $k_{V_{h}}$, respectively.

**Figure 6:**
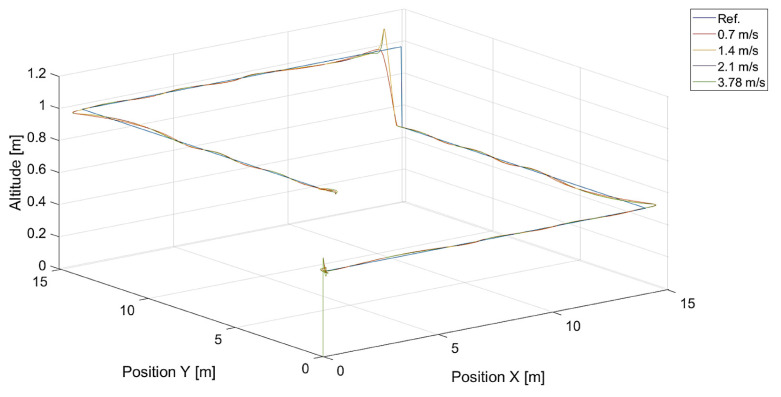
Plot of trajectories of the point (a rectangle) to be tracked and the quadcopter for different values of $V_{max}$. Ref. is the reference trajectory of the tracked point; values of $V_{max}$ are 0.7 m/s, 1.4 m/s, 2.1 m/s, and 3.78 m/s.

**Figure 7:**
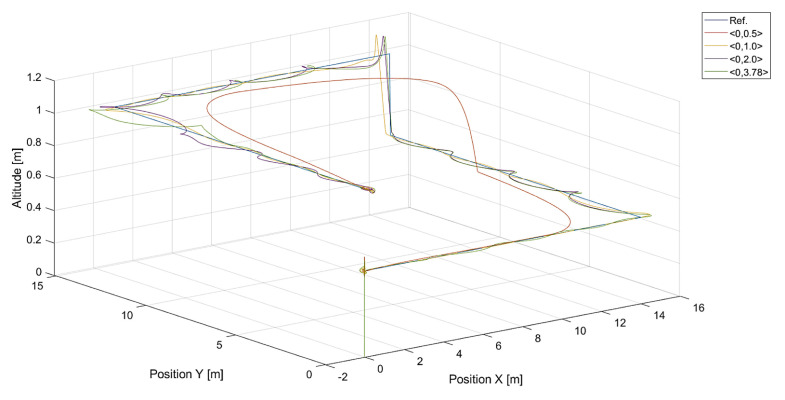
Plot of trajectories of the point (a rectangle) to be tracked and the quadcopter for different values of the upper limit of $\left|V_{Cr}\left(x,y\right)\right|$: 0.5, 1.0, 2.0, and 3.78. The value of $V_{max}$ was 3.78.

In Figure 6, there are flight trajectories for different values of $V_{max}$, which decide the maximum relative speed between the tracked point and the quadcopter and how fast the tracking error is minimized. It can easily be noticed that there is no significant difference between trajectories for $V_{max}$ values equal to 1.4, 2.1 m/s, and 3.78 m/s. Of course, these values are greater than the speed of the tracked point, 0.7 m/s. If we consider that the speed of a chasing vehicle should be greater than the speed of the pursued, and the shape of the reference trajectory has 90-degree turns, it can be concluded that the desired speed of the quadcopter from Equation (14) will always satisfy this requirement. Only differences in vertical position tracking between trajectories for the mentioned values of $V_{max}$ greater than 1.4 m/s can be observed, and this means that horizontal trajectory tracking for these values is limited by Equations (17) and (18), where the $\left|V_{Cr}\left(x,y\right)\right|$ range was set to <0,1.0>. Despite this, overshoots in longitudinal and horizontal speed control can still be observed. To illustrate this effect, trajectories from simulations with different upper limits of $\left|V_{Cr}\left(x,y\right)\right|$ are presented in Figure 7.

**Figure 8:**
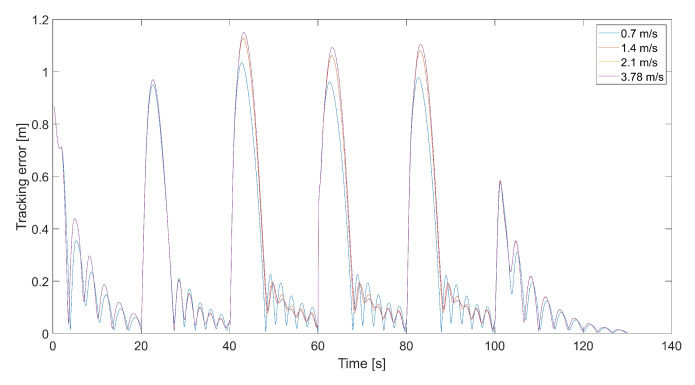
Plot of the tracking error for the quadcopter’s flights and different values of $V_{max}$, i.e., 0.7 m/s, 1.4 m/s, 2.1 m/s, and 3.78 m/s ($k_{\dot{\Psi }}=1.0$, $k_{V_{h}}=1.0$).

Figure 8 presents plots of the tracking error at different maximum values of $V_{max}$. Maximums of the tracking error appear at the moment of 90-degree turns and then decrease almost to zero. When the tracking error approaches zero, the length of the velocity vector *V* decreases, and the quadcopter slows down, guided only by $V_{T}$, and the tracking error increments. This happens periodically; thus, it is a good idea to design a dead zone around the tracked point to prevent a reduction in the quadcopter’s speed near the tracked point $V_{T}$. Inside the dead zone, the velocity vector $V_{C}$ could be equal to the velocity vector of the tracked point. A disadvantage of the dead zone is that the quadcopter never reaches the tracked position, and the minimum tracking error in the steady state depends on the radius of the dead zone. Interestingly, the maximum tracking error is greater for higher values of $V_{max}$. This means that a higher relative speed does not guarantee lower values of the tracking error because it can cause an overshoot in position tracking, as can be seen in Figure 6. On the other hand, for the value of 0.7 m/s, there are higher amplitudes of tracking error oscillations when the quadcopter almost reaches the position of the tracked point.

**Figure 9:**
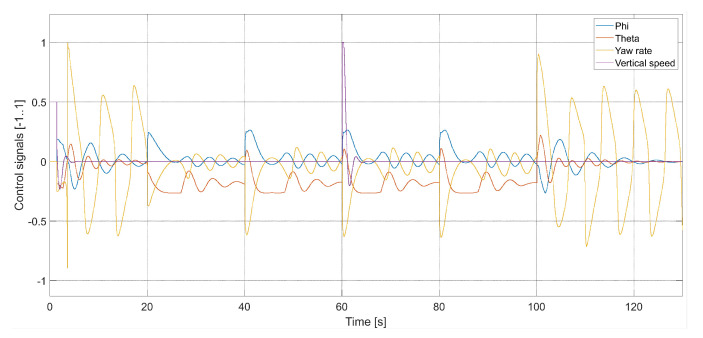
Plot of the quadcopter’s control signals ($V_{max}=3.78$, $k_{\dot{\Psi }}=1.0$, $k_{V_{h}}=1.0$), i.e., phi—desired value of roll $\phi_{C}$, theta—desired value of pitch $\theta_{C}$, yaw rate—desired value of ${\dot{\Psi }}_{C}$, and vertical speed—desired value of $V_{h}$.

Figure 9 presents plots of control signals for the flight with $V_{max}=3.78$ m/s.

**Figure 10:**
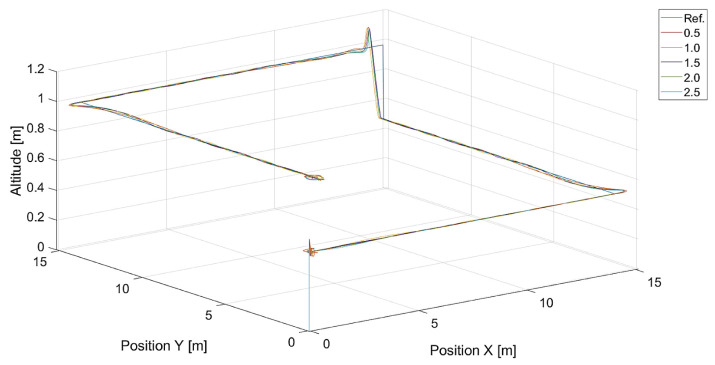
Plot of trajectories of the point to be tracked (a rectangle) and the quadcopter for different values of $k_{\dot{\Psi }}$ ($V_{max}=3.78$, $k_{V_{h}}=1.0$). Ref. is the reference trajectory of the tracked point; values of $k_{\dot{\Psi }}$ are 0.5, 1.0, 1.5, and 2.0.

**Figure 11:**
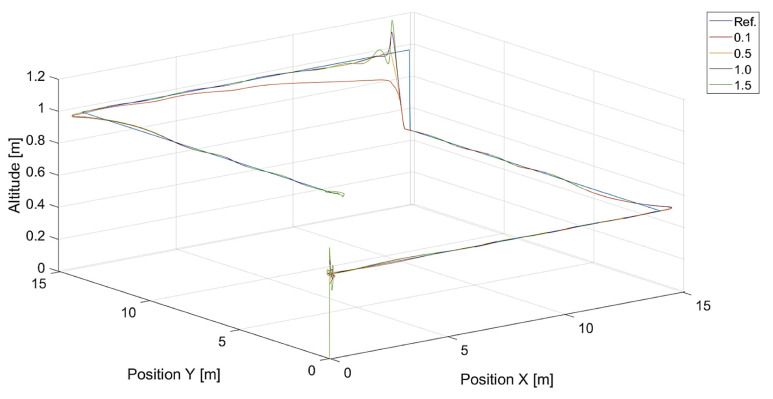
Plot of trajectories of the point to be tracked (a rectangle) and the quadcopter for different values of $k_{V_{h}}$ ($V_{max}=3.78$, $k_{\dot{\Psi }}=1.0$). Ref. is a reference trajectory of the tracked point; values of $k_{V_{h}}$ are 0.1, 0.5, 1.0, and 1.5.

In turn, Figure 10 and Figure 11 present flight trajectories for different values of $k_{\dot{\Psi }}$ and $k_{V_{h}}$, respectively. Any value greater than 0.5, either for $k_{\dot{\Psi }}$ or for $k_{V_{h}}$, results in an increment in the displacement of the quadcopter’s position with respect to the reference trajectory. The lowest value of $k_{V_{h}}$, i.e., 0.1, introduces inertia into the response of the vertical control.

**Figure 12:**
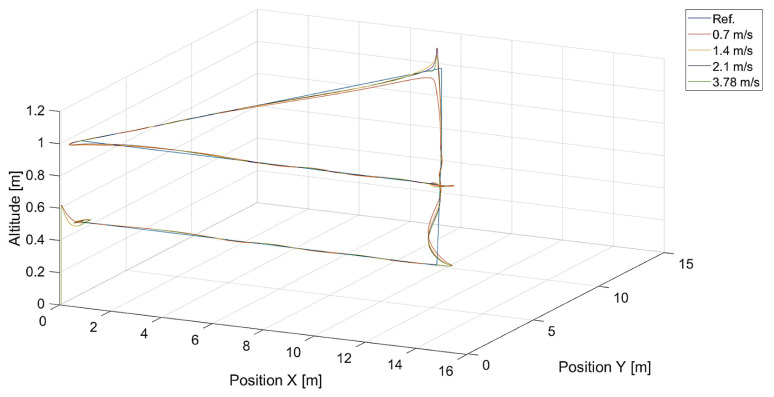
Plot of trajectories of the point to be tracked (a triangle) and the quadcopter for different values of $V_{max}$. Ref. is the reference trajectory of the tracked point; values of $V_{max}$ are 0.7 m/s, 1.4 m/s, 2.1 m/s, and 3.78 m/s.

**Figure 13:**
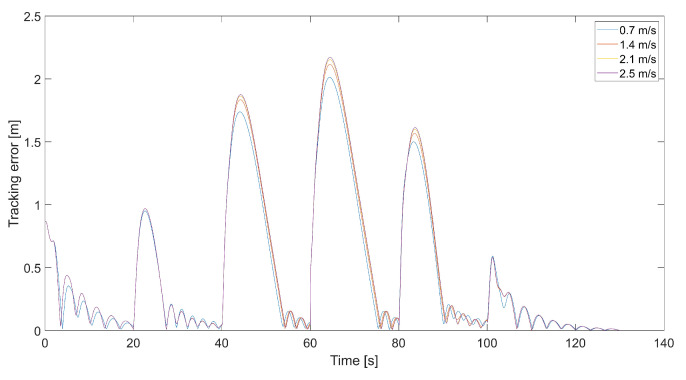
Plot of the tracking error for the quadcopter’s flights and different values of $V_{max}$, i.e., 0.7 m/s, 1.4 m/s, 2.1 m/s, and 3.78 m/s ($k_{\dot{\Psi }}=1.0$, $k_{V_{h}}=1.0$).

**Figure 14:**
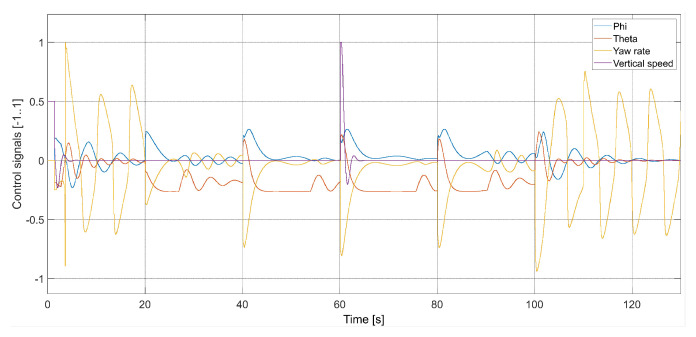
Plot of the quadcopter’s control signals ($V_{max}=3.78$, $k_{\dot{\Psi }}=1.0$, $k_{V_{h}}=1.0$), i.e., phi—desired value of roll $\phi_{C}$, theta—desired value of pitch $\theta_{C}$, yaw rate—desired value of ${\dot{\Psi }}_{C}$, and vertical speed—desired value of $V_{h}$.

**Figure 15:**
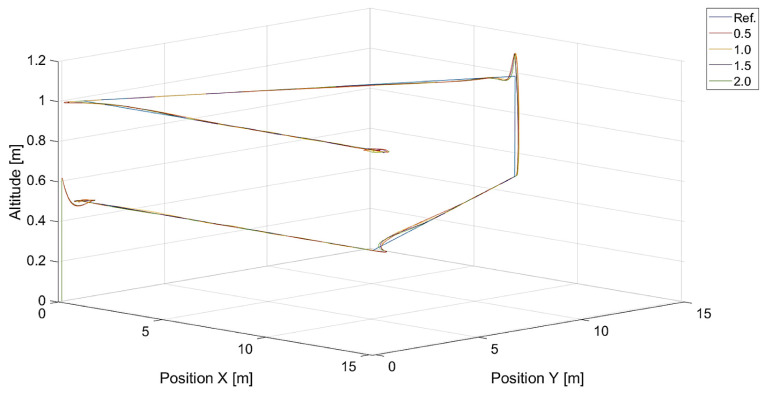
Plot of trajectories of the point to be tracked (a triangle) and the quadcopter for different values of $k_{\dot{\Psi }}$ ($V_{max}=3.78$, $k_{V_{h}}=1.0$). Ref. is the reference trajectory of the tracked point; values of $k_{\dot{\Psi }}$ are 0.5, 1.0, 1.5, and 2.0.

**Figure 16:**
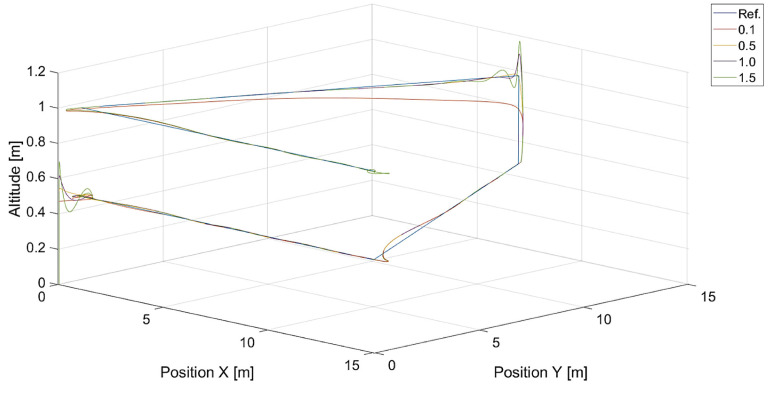
Plot of trajectories of the point to be tracked (a triangle) and the quadcopter for different values of $k_{V_{h}}$ ($V_{max}=3.78$, $k_{\dot{\Psi }}=1.0$). Ref. is the reference trajectory of the tracked point; values of $k_{V_{h}}$ are 0.1, 0.5, 1.0, and 1.5.

**Figure 17:**
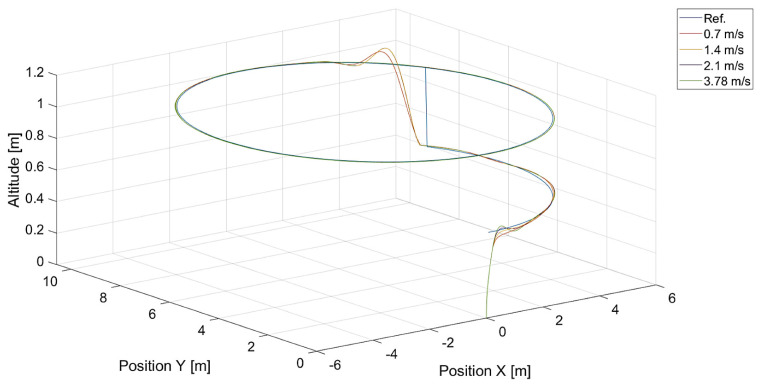
Plot of trajectories of the point to be tracked (a circle) and the quadcopter for different values of $V_{max}$. Ref. is the reference trajectory of the tracked point; values of $V_{max}$ are 0.7 m/s, 1.4 m/s, 2.1 m/s, and 3.78 m/s.

**Figure 18:**
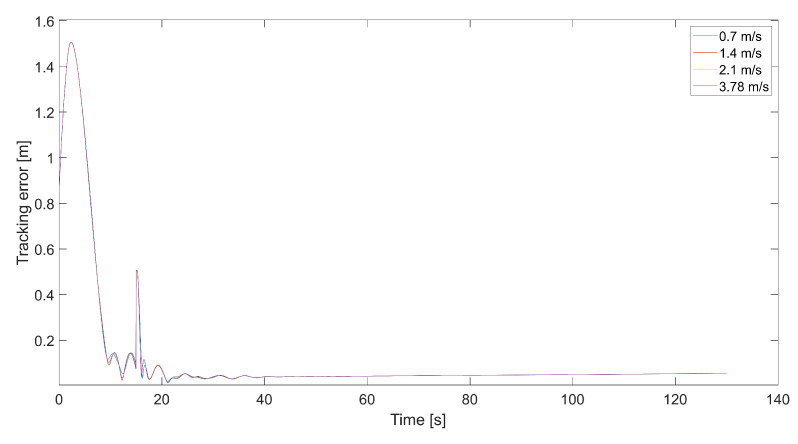
Plot of the tracking error for the quadcopter’s flights and different values of $V_{max}$, i.e., 0.7 m/s, 1.4 m/s, 2.1 m/s, and 3.78 m/s ($k_{\dot{\Psi }}=1.0$, $k_{V_{h}}=1.0$).

**Figure 19:**
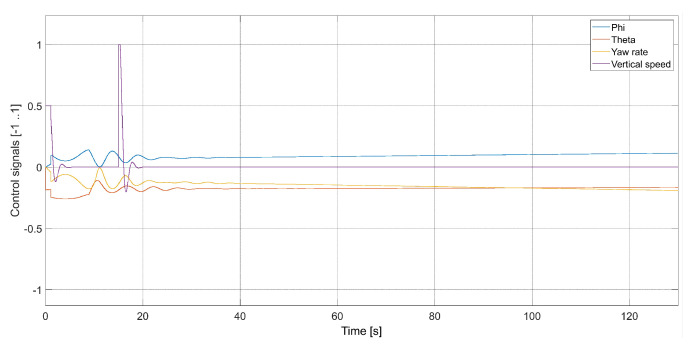
Plot of the quadcopter’s control signals ($V_{max}=3.78$, $k_{\dot{\Psi }}=1.0$, $k_{V_{h}}=1.0$), i.e., phi—desired value of roll $\phi_{C}$, theta—desired value of pitch $\theta_{C}$, yaw rate—desired value of ${\dot{\Psi }}_{C}$, and vertical speed—desired value of $V_{h}$.

**Figure 20:**
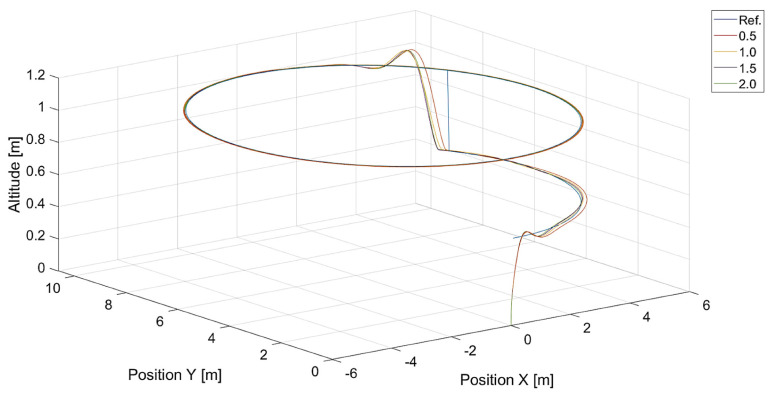
Plot of trajectories of the point to be tracked (a circle) and the quadcopter for different values of $k_{\dot{\Psi }}$ ($V_{max}=3.78$, $k_{V_{h}}=1.0$). Ref. is the reference trajectory of the tracked point; values of $k_{\dot{\Psi }}$ are 0.5, 1.0, 1.5, and 2.0.

**Figure 21:**
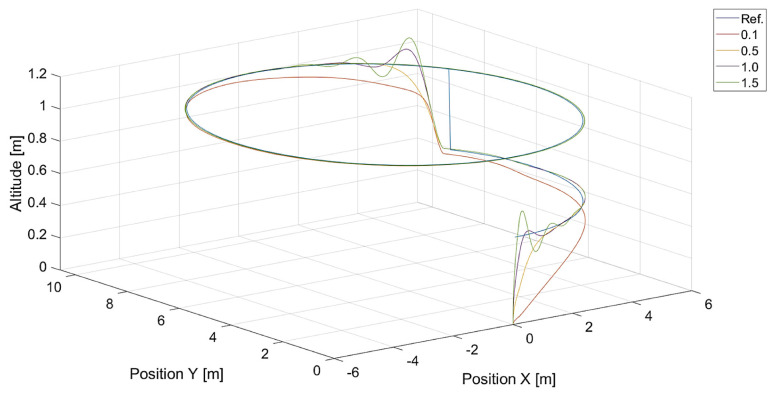
Plot of trajectories of the point to be tracked (a circle) and the quadcopter for different values of $k_{V_{h}}$ ($V_{max}=3.78$, $k_{\dot{\Psi }}=1.0$). Ref. is the reference trajectory of the tracked point; values of $k_{V_{h}}$ are 0.1, 0.5, 1.0, and 1.5.

Figure 12, Figure 13, Figure 14, Figure 15, Figure 16, Figure 17, Figure 18, Figure 19, Figure 20 and Figure 21 present similar plots for the triangle and circular reference trajectories in sequence. The impact of the gradient length’s saturation $V_{max}$ and coefficients $k_{\dot{\Psi }}$ and $k_{V_{h}}$ on the tracking error is also identical. In the cases of the rectangle and triangle trajectories, we can notice that the quadcopter flies around the initial position of the tracked point randomly before it starts to move. It is because of that that the quadcopter’s control is designed to track a moving point, and so that the quadcopter’s front is facing it. Hence, the flight of a quadcopter looks like the flight of an aircraft which is only able to circle a stationary point.

**Figure 22:**
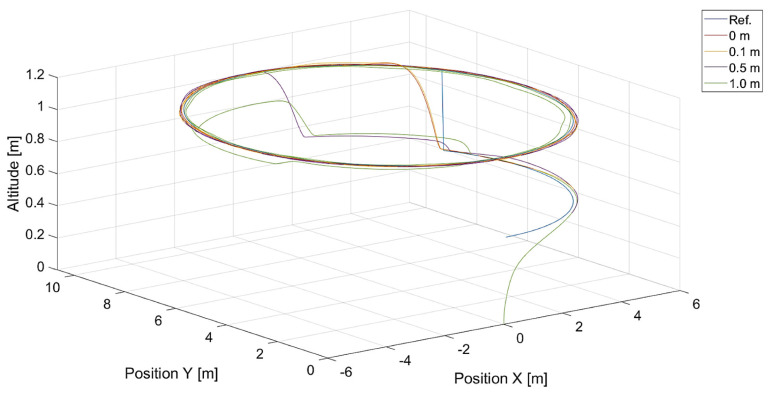
Plot of trajectories of the point to be tracked and the quadcopter for different radii of the dead zone around the tracked point ($V_{max}=3.78$, $k_{V_{h}}=1.0,k_{\dot{\Psi }}=1.0$).

The last Figure 22 visualizes trajectories of the quadcopter when the tracked trajectory is a circle, and there is a dead zone with different radii around the tracked point. As mentioned, the dead zone is an area inside which the relative speed derived from the artificial potential field should be zero. Then, the quadcopter is controlled only by vector $V_{T}$, and its movement is synchronized with the tracked point. An excessively long range of the dead zone results in the impossibility of trajectory tracking, and we can observe a varying displacement between the reference and the quadcopter’s trajectory. When the quadcopter is inside the dead zone, it flies with the same speed vector $V_{T}$ as the tracked point. Thus, it is unable to minimize vertical displacement. If the tracking error increases, and the quadcopter is outside the dead zone, it starts to use the sum of vectors *V* and $V_{T}$ and climbs toward the altitude of the tracked point. That is why we can observe a few changes in altitude made by the quadcopter instead of a single change.

**Figure 23:**
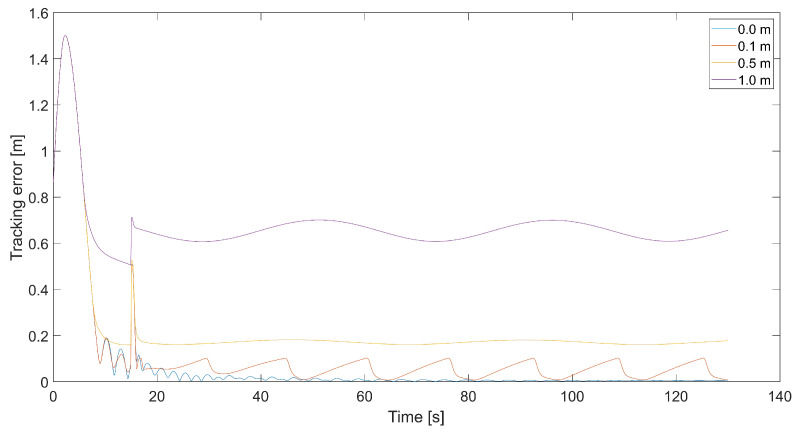
Plot of the tracking error for different radii of the dead zone around the tracked point ($V_{max}=3.78$, $k_{V_{h}}=1.0,\;k_{\dot{\Psi }}=1.0$).

Figure 23 presents plots of the tracking error for the trajectories from Figure 22. The tracking error’s magnitude correlates with the dead zone’s radius. Still, the decrement in the radius increases the frequency of tracking error oscillations when it approaches zero.

**Figure 24:**
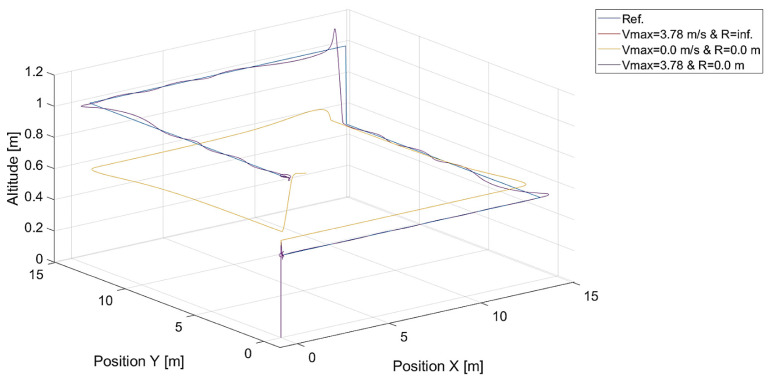
Plot of trajectories of the point to be tracked and the quadcopter for a radius of the dead zone around the tracked point equal to infinity ($R=inf.,V_{max}=3.78$ m/s), saturation of relative speed $V_{max}=0.0$ (R=0.0 m), and the case when the radius is zero (R=0.0 m) and $V_{max}=3.78$ ($k_{V_{h}}=1.0,k_{\dot{\Psi }}=1.0$).

Finally, it can be tested what happens when the radius of the dead zone is infinite or the maximum length of the gradient, i.e., $V_{h}$, is zero. These two independent cases should be equivalent and give the same results as in Figure 24.

**Figure 25:**
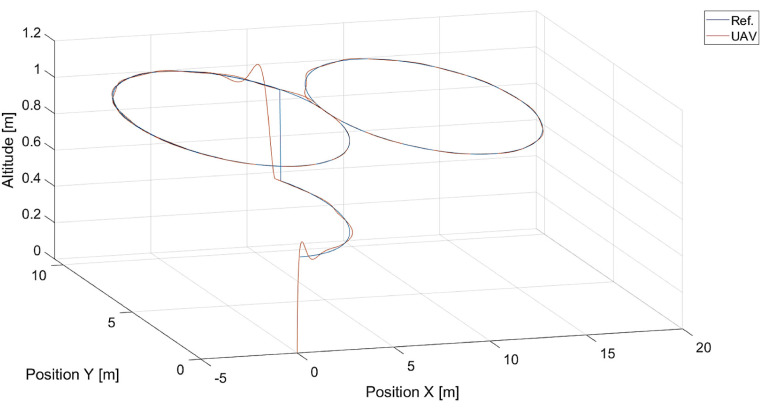
Plot of trajectories of the point to be tracked and the quadcopter (R=0.0 m, $V_{max}=3.78$ m/s, $k_{V_{h}}=1.0,k_{\dot{\Psi }}=1.0$).

The last Figure 25 presents results of moving point tracking for a more challenging shape of the reference trajectory, i.e., eight-shaped.

## 5. Conclusions

Waypoint navigation allows unmanned aerial vehicles to perform flights along a trajectory composed of lines and arcs based on predefined points. Flying through complicated trajectory curves requires the design of specific flight controls that predetermine a flight route using s-curves or cubic curves. These algorithms cannot be used to track a moving object whose trajectory is unknown, even if it is possible to determine its relative position onboard in real time, i.e., a leader–follower framework. The artificial potential field is a simple, well-known method to guide a UAV toward a goal among obstacles whose locations are known. This method can also be applied in tasks of position tracking of other moving objects, whose position can be determined, or to track a complicated trajectory generated analytically as a series of points in real time. This article presented a method that used an artificial potential field to compose a control velocity vector that made the Parrot quadcopter able to fly through a generated trajectory. The control velocity vector $V_{C}$ was composed of two parts, i.e., velocity vector *V* obtained from the potential gradient and vector $V_{T}$, the velocity of the tracked point calculated as the derivative of its position.

Simulation results presented the proposed method’s capabilities and proved that the quadcopter could track the shape of the generated trajectory with a tracking error below 0.2–0.5 m. Only during sharp turns of about 90 degrees and greater did the tracking error exceed a value of 1 m, but the shape of the flight trajectory was slightly distorted by overregulation. Tuning gains of vertical and yaw controls, i.e., $k_{V_{h}}$ and $k_{\dot{\Psi }}$, made it possible to reduce that distortion. Based on Figure 11, Figure 12, Figure 15, Figure 16, Figure 20 and Figure 21, it can be stated that values of these gains equal to about 0.5 eliminate or reduce the overregulation significantly. Observing the impact of $V_{max}$ on flight trajectories in all cases of reference trajectory shape, it can be concluded that the maximum relative speed does not have to be greater than the speed of the tracked point. For higher values of $V_{max}$ saturation, the overregulation (mainly vertically) increases along with the tracking error during turns. Determining the dead zone around the tracked point helps to reduce oscillations of the tracking error near the tracked point but also increases the tracking error in the steady state.

Reducing the maximum relative speed defined by $V_{max}$ to zero or setting the infinite radius of the dead zone results in a situation where the quadcopter is unable to minimize the tracking error. Therefore, the radius of the dead zone should be a compromise between the allowable magnitude of the tracking error and the unwanted effect of oscillations when the quadcopter is near the tracked point.

The main limit of the proposed method is a high dependence on the knowledge of the actual position and speed of the tracked target. Therefore, specialized vision sensors with a wide field of view must be utilized in experiments with real drones to determine the relative position of the tracked target independently from the drone’s relative orientation, and this could be challenging. In indoor experiments, a local positioning system like Optitrack or UWB-based (ultrawideband) system successfully solves the problem. Generating a virtual point to be tracked is another possibility of verifying the algorithm in the real world.

Although the presented results are satisfactory, instead of constant values of coefficients $V_{max}$, $k_{V_{h}}$, and $k_{\dot{\Psi }}$, which are only proportional gains, it is possible to implement PID regulators to improve response to the altitude, heading, and linear speed changes of the tracked point’s movement. This problem and hardware-in-the-loop tests will be the next step in our research. The next area of further research activity will be developing control algorithms based on neural networks, AI, or deep learning, which will use simulated or recorded signals generated by the artificial-potential-field controller for different trajectory shapes as a learning data set. Experimental tests with the use of a local positioning system like OptiTrack or UWB-based system and a mobile robot as a target are also planned.

## Data Availability

Data are contained within the article.
